# Psychophysiological insights and user perspectives: enhancing police de-escalation skills through full-body VR training

**DOI:** 10.3389/fpsyg.2024.1390677

**Published:** 2024-09-04

**Authors:** John E. Muñoz, Jennifer A. Lavoie, Alan T. Pope

**Affiliations:** ^1^Department of Liberal Arts, Wilfrid Laurier University, Brantford, ON, Canada; ^2^Department of Systems Design Engineering and The Games Institute, University of Waterloo, Waterloo, ON, Canada; ^3^Departments of Criminology and Psychology, Wilfrid Laurier University, Brantford, ON, Canada; ^4^NASA Langley Research Center, Hampton, VA, United States

**Keywords:** virtual reality, police training, mental health crisis, psychophysiology, user experience, de-escalation

## Abstract

In recent years, Virtual Reality (VR) has emerged as a promising tool for enhancing training responses in high-stress professions, notably among police officers. This study investigates the psychophysiological responses and subjective user experience of active police officers undergoing Mental Health Crisis Response (MHCR) training using an immersive full-body VR system. A total of 10 active police officers with Special Weapons and Tactics (SWAT) training participated in our controlled study. Officers independently took part in one VR training session lasting 7–12 min involving an avatar in crisis portrayed by an actor. Officers wore integrated cardiovascular and electrodermal activity measurement devices for physiological monitoring. VR user experience aspects such as induced symptoms or game mechanics were investigated upon completing the training, aiming to evaluate the officer’s perceptions of the technology. We used the DePICT™ scale to evaluate the de-escalation skills of officers, coded by a research professional. Our findings revealed significant differences in heart rate and heart rate variability responses between baseline and VR scenario immersion, suggesting heightened stress regulation during the MHCR simulation using full-body VR. Arousal measurements also revealed measurable responses during the training in VR. Additionally, the user experience assessment indicated a positive reception to the VR training, with minimal VR-induced symptoms. A “Defensive-Dynamics-Dichotomy” was revealed highlighting dominant autonomic responses linked to defensive actions (e.g., officers who drew a weapon; those who kept their weapons holstered) and their respective implications for stress management and cognitive function. A unique constellation of de-escalation skills was revealed among officers who relied on weapons relative to those who did not, to resolve the scenario. The study highlighted the perceived utility of physiological monitoring technologies in enhancing police training outcomes. In conclusion, our research underscores the potential of VR as an effective tool for de-escalation training following MHCR simulated scenarios among active police officers, offering insights into its psychophysiological impact and user experience. The findings contribute to improving our understanding of the physiology associated with decision-making in police officers to draw a weapon, emphasizing the role of advanced simulation and physiological monitoring technology in developing evidence-based training programs for public safety.

## 1 Introduction

In high-stakes law enforcement training, the ability to respond effectively to mental health crises is as critical as it is challenging. Traditional training methods for mental health crisis response have often fallen short in preparing officers for the dynamic and emotionally charged nature of these encounters (Dubé, 2016). Evidence suggests that police officers receive insufficient training in responding to mental health calls for service and have training gaps in fundamental de-escalation skills (Coleman and Cotton, 2014). Consequently, police responses to people with mental illness tend to result in higher use-of-force incidents (Morabito et al., 2018; Hallett et al., 2021), including lethal force (Lorey and Fegert, 2021), compared to those without reported mental illness. Initiatives towards creating more informed and contextualized training for law enforcement officers to respond to mental health crises have been piloted, revealing promising results in de-escalation skills improvement and potential for reducing unnecessary force in police interactions (Lavoie et al., 2022, 2023). Adequately designed training is necessary for developing the best possible practices in policing when addressing individuals with mental health disorders (Lavoie et al., 2022). The advent of technological tools, such as Virtual Reality (VR), offers a new horizon in police training, promising an immersive experience that could bridge the gap between theoretical knowledge and real-world application (Giessing, 2021). The use of virtual reality (VR) training for police officers has gained attention as a potential tool for enhancing training responses under stress (Giessing, 2021). VR offers multiple benefits such as the possibility to create limitless and highly personalized scenarios for training, enhanced safety in a controlled training environment, the realism and deep immersivity of the simulations, and the possibility of collecting rich data during the interaction for a debrief session with the trainees. VR has been recognized as one of the most powerful solutions to improve training in first responders and provide an effective way of preparing frontline police officers, particularly for the demands they face in the field (Alanis and Pyram, 2022). Mental health crisis scenario training has been found to be as effective in improving de-escalation competency acquisition among police officers using VR platforms as more traditional live-action formats (Lavoie et al., 2023).

While previous studies have investigated the physical and psychological training responses in police officers, there is a lack of research on the psychophysiological responses of VR training for mental health crisis response (MHCR) training among other training domains, in active police officers. Psychophysiology is the scientific study of the interactions between psychological processes and physiological systems (Cacioppo et al., 2007). Central to this field are the sympathetic and parasympathetic nervous systems, which form the autonomic nervous system. The sympathetic nervous system orchestrates the “fight-or-flight” response, preparing the body for action in stressful situations by increasing heart rate, blood pressure, and respiration, among other functions. Conversely, the parasympathetic nervous system facilitates the “rest-and-digest” response, promoting relaxation and energy conservation by decreasing heart rate and stimulating digestion (Cacioppo et al., 2007). Several scientific articles have studied the physiological responses of police officers in various situations, including general-duty police encounters (Baldwin et al., 2019) and the psychological impact of COVID-19 on police officers (Tehrani, 2022). Studying psychophysiological responses of police officers to VR scenario training is of value since simulation technologies are now widely used in many police departments (Andersen et al., 2020; Kleygrewe, 2023) and there is little evidence of its effectiveness in generating measurable body responses, which could explain associated psychological phenomena such as stress (Giannakakis et al., 2019). This is especially true for police training to respond to mental health crises, where subconscious elements such as the officer’s implicit biases, perception of threat, or health-related components such as fitness levels, can dramatically impact an officer’s performance during these calls. Investigating those responses could potentially reveal training gaps in relevant aspects such as decision-making, situational awareness, self-regulation, stress management, and cognitive readiness among many others. Since stress significantly impacts police performance, understanding the physiological responses to stress is crucial for developing effective training programs that prepare officers for real-world situations (Di Nota and Huhta, 2019).

This study seeks to fill that void by examining the psychophysiological responses and subjective experiences of active police officers engaged in VR-based mental health crisis training scenarios. Utilizing an immersive full-body VR system, the research investigates the intricate interplay between measured physiological arousal, stress regulation, and the perceived usefulness and induced symptoms (e.g., nausea) of VR training scenarios. Next, we will provide a summary of the research evidence of studying body responses of police officers using VR technologies for a variety of simulations including firearms and de-escalation training.

## 2 Related work

Previous studies have investigated physical and psychological training responses in VR, including heart rate (HR), level of physical activity, mental effort, and perceived stress involving law enforcement officers (Muñoz et al., 2020; Giessing, 2021). Research with a stressful VR training scenario demonstrated that a stress dashboard, based on bio-signal data, provided trainers with actionable insights via assessment of trainees’ cognitive states (Zechner et al., 2023). An analysis of the strengths, weaknesses, opportunities, and threats (SWOT) associated with the use of VR in scenario-based stress training in police settings was conducted, with one recommendation being the study of real-time psychophysiological stress markers during realistic VR scenarios (Giessing, 2021). In a VR firearms training routine, trained police officers exhibited different brainwave activity and heart rate variability (HRV) parameters between resting vs. active shooting conditions, providing a basis for a psychophysiological model for firearms training (Muñoz et al., 2020). The study used a combination of multiple physiological signals describing sympathetic (fight and flight) and parasympathetic (rest and digest) responses analyzed with behavioral data from the simulation and subjective ratings collected after the simulation. Similar results were found in a firearm simulation scenario in VR involving civilians with minimal levels of gun training (Muñoz et al., 2019). Another study investigated the physiological responses of police officers to audio-visual stress cues in VR based on HRV and HR responses; in particular, a stress dashboard was developed for monitoring trainees’ stress levels during the training for them to be included in a debriefing (Zechner et al., 2023). The combination of VR and real-time physiological feedback (e.g., biofeedback) presents opportunities to improve self-regulation and stress management training since officers could be more aware of their body signals and adapt their reactions accordingly (De Witte et al., 2019). Previous studies have shown clear and objective evidence of the importance of physiologically focused interventions in reducing errors in lethal force decision-making in active police officers in Canada (Andersen et al., 2018).

A study examined how officers’ HR varied with the phase (e.g., encountering a subject) of a general duty call for service as well as incident factors (e.g., use-of-force) (Hickman et al., 2011). In comparing scenario-based training in VR vs. real life, police officers experienced similar average levels of HR and levels of perceived stress with differences in maximum heartbeats attributable to differences in the physical activity involved (Kleygrewe et al., 2023). This study showed how VR is capable of creating measurable physiological responses, comparable to the ones obtained in conventional simulation-based training scenarios. Another study was conducted during a critical incident simulation. Police officers’ verbal and non-verbal communication and tactical skills, all rated by experts, were not affected by elevated HR or change in circulating cortisol measured before and after the simulation. Better overall performance, tactical performance, and nonverbal communication, but worse verbal communication, were associated with a greater increase in circulating antithrombin, a natural anticoagulant countermeasure to stress-induced blood clotting (Arble et al., 2019). The results indicated that the effects of physiological arousal on performance varied, with some levels of arousal potentially enhancing performance while others may impair it, highlighting the complex relationship between stress and performance in high-stakes situations (Arble et al., 2019). Whereas the study of human behavior, emotions (Marín-Morales et al., 2020), and psychophysiological responses using VR have been widely used to investigate stress reactivity across different populations (van Dammen et al., 2022), it is surprising to find that, to the best of our knowledge, no studies have been conducted with active police officers using physiological sensors to understand the underlying mechanisms of stress and self-regulation during de-escalation training using VR. Studying psychophysiological responses to mental health crisis simulations and other police training in VR could provide valuable insights into the effectiveness of VR-scenario-based training in key skills such as stress management and self-regulation, therefore contributing to the development of evidence-based training programs supported with advanced simulation technology. Therefore, this study has two main objectives:

- Measuring the sympathetic activation responses of a group of trained police officers while interacting with a full-body immersive VR simulation using a mental health crisis scenario.
- Investigate the perceived user experience and de-escalation skills of police officers after being immersed in a mental health crisis scenario involving an avatar played by a responsive live actor.

The results of this study aim to contribute significantly to the field of law enforcement training by providing empirical data on the physiological effects of VR training and its relationship to observed de-escalation competencies when responding to mental health crisis calls. The findings could lead to more effective training protocols, better-prepared officers, and ultimately, safer and more desirable outcomes for both police and the public in mental health crises.

## 3 Methodology

### 3.1 System setup

**Figure 1:**
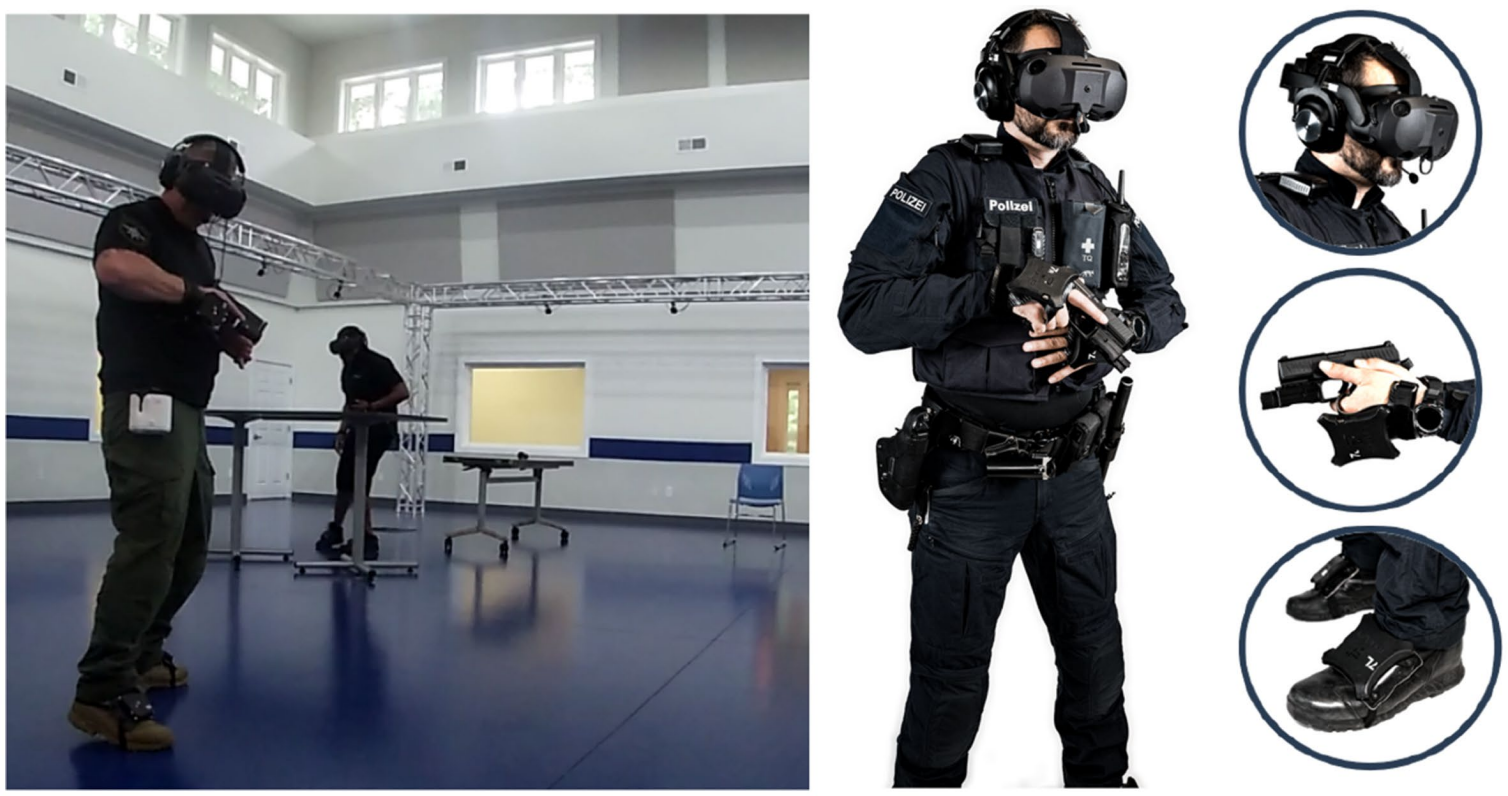
**(Left)** Training center with physical prompts used for the VR scenario. **(Right)** Police officer and VR equipment used for the study.

A full-body immersive VR setup was used to create a realistic scenario for the mental health crisis simulation. The Refense^1^ system uses motion capture cameras and trackers which allow officers to have a realistic and immersive experience. Officers are equipped with a VR HMD (HTC Vive Focus 3), and five motion capture trackers positioned on both arms, feet, and back. A microphone and audio were provided via over-ear headphones, whereas an integrated replica handgun was worn in a plastic holster around the hips. A feedback belt that creates electrical shocks was also attached to the officer’s stomach to simulate potential attacks and shots. Figure 1 shows the VR equipment used in the Refense system. The VR system is set up in a 15 × 15 m area. Two tables and one chair were physically located to match virtual objects and add realism. Two people were used to simulate the mental health call or service (i) a dispatcher agent who communicated with the officer when called, (ii) a live actor wearing similar VR gear (no weapons or belt) to portray the person with a mental health crisis.

### 3.2 The mental health crisis simulation

**Figure 2:**
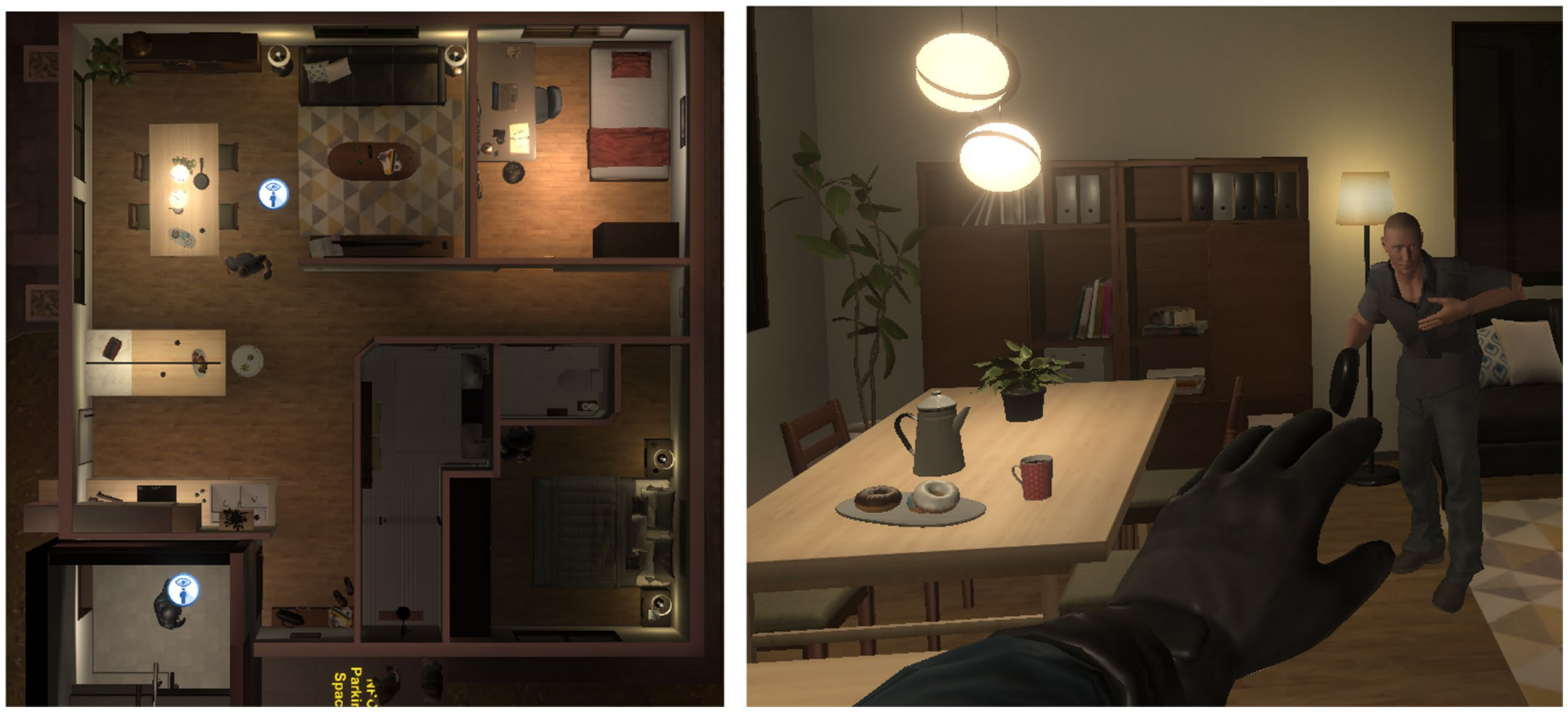
**(Left)** Overhead view of the VR scenario. **(Right)** Officer’s first-person view. Adapted with permission from Refense.

The Officers were instructed to use their police training to respond to a 10-min scenario simulation in VR. Officers listened to a brief dispatch describing a person in crisis who had summoned police to his private residence for assistance. Officers completed the VR scenario individually. The character in crisis in this scenario was portrayed as a white male, his early 30s, with rib injuries (see Figure 2). In the scenario narrative, the character displays Post-Traumatic Stress Disorder (PTSD) symptoms heightened by cannabis intoxication. He is in crisis believing that the people responsible for invading his home a few weeks ago have returned to attack him. He is irritable (yelling, non-cooperative), fearful (shortness of breath, weeping), and hypervigilant (continuously looking out of the window, asking the officer if anyone is there). Officers began the simulation at the virtual front door of the residence, and the scenario took place inside a hallway, kitchen, dining area, and living room in the residence. The character in crisis was designed to demonstrate de-escalation behaviors in response to officers engaging in de-escalation strategies (willingness to help, active listening, reassurance, offering choices), and escalation behaviors when officers were not employing these strategies (drawing a firearm, dismissing concerns, yelling, interrupting, returning to “triggers”). The scenario was divided into three main stages: (i) arrival, which occurred during the first minute when Officers were confronted with the character in crisis yelling at them after opening the door, and initial survey of the environment for safety risks, (ii) conflict escalation with a blunt object based on pre-determined triggers, the character in crisis escalates and creates a safety risk by wielding a frying pan for protection, (iii) de-escalation and problem-solving, after reassurance of his safety, the character in crisis relinquishes the frying pan and will agree to sit down. In response to questions, in a de-escalated state, he will provide additional cues about his situation (intoxication, recent loss) and desired possible resolutions (call a close friend). The scenario used in the study is derived from a series of evaluation scenarios that were developed and produced through a community co-design approach leveraging expertise and values from community stakeholders as well as police instructors (Lavoie et al., 2022).

Subject matter experts from across major domains took part in focus groups with outputs being (i) the development of a novel conceptual approach to police responses to people in a mental health crisis (relational policing approach), (ii) a scenario-based experiential learning training program that offers structured opportunities for officer trainees to rehearse de-escalation, officer safety, and communication strategies across a diverse range of carefully crafted high-fidelity crisis scenarios depicting a variety of environments, characters, behaviors and situations. The program centers on the acquisition of core competencies related to relational policing, de-escalation, and mental health crisis response. The training was designed to first deliver the curriculum by bringing learners through a spectrum of authentic crisis scenarios and using a group problem-based learning approach. Officers are then allowed to test their abilities to effectively communicate, de-escalate, maintain safety, and make ethical decisions under stress. After scenario completion, officers debriefed for approximately 10 min with researchers and a police instructor, who observed and was always present during the simulation training.

The study was conducted in collaboration with a local police department in Virginia (USA). A total of 10 active police officers with special weapons and tactics (SWAT) training were invited to be part of the study. The recruitment was internally conducted by the instructor at the police department who offered a voluntary VR training session to the active police officers with SWAT training at the department. SWAT members were selected due to schedule convenience with other tactical training happening on the same day. Most of the officers had not used VR prior to the study (see Table 1).

**Table 1:** Demographics of the officers.

**User ID**	**Gender**	**Age range**	**Ethnicity**	**Education**	**Years of service**	**LEO trainer**	**Military veteran**	**Experience with MHC**	**Use VR before**
1	Male	45–54	White	Some college, no degree	11–16 years	Yes	No	No	Yes
2	Male	35–44	White	Some college, no degree	2–4 years	No	No	No	No
3	Male	35–44	White	Bachelor’s degree (e.g., BA, BS)	11–16 years	Yes	No	Yes	No
4	Male	25–34	White	Some college, no degree	5–10 years	Yes	No	Yes	No
5	Male	25–34	White	Some college, no degree	11–16 years	Yes	Yes	Yes	No
6	Male	25–34	Black or African American	High school degree or equivalent (e.g., GED)	2–4 years	No	No	No	No
7	Male	45–54	Black or African American	Some college, no degree	17+	Yes	Yes	Yes	Yes
8	Male	45–54	Black or African American	Bachelor’s degree (e.g., BA, BS)	17+	Yes	Yes	Yes	Yes
9	Male	35–44	White	Some college, no degree	5–10 years	Yes	No	No	No
10	Male	25–34	White	Bachelor’s degree (e.g., BA, BS)	11–16 years	Yes	No	Yes	No

LEO, law enforcement officers.

### 3.3 Outcome measures

#### 3.3.1 Physiological metrics

Several wearable physiological sensors were used to collect the body responses of officers during the interaction.

##### 3.3.1.1 Cardiovascular

The chest strap sensor Polar H10 was used to record the cardiovascular responses of police officers. It includes built-in algorithms that were used to filter and calculate ECG parameters needed to compute heartbeats and HRV parameters while streaming data at 1 Hz. The sensor and proprietary algorithms have been optimized to accurately capture the responses of officers during movement (Plews et al., 2017). The main variable computed is called the R-to-R intervals (RRI) with units of 1/1024 s. HR and RRIs are captured using a proprietary and open-source tool connected to an Android tablet which saves the data locally via Bluetooth Low Energy (Quintero et al., 2021). The HRV analysis included multiple metrics that express overall autonomic nervous system activity (Ernst, 2014). The Neurokit2 Python library was used to conduct the HRV analysis (Makowski et al., 2021). Extracted features include the standard deviation of the RRIs (SDNN) and the root mean square of successive differences (RMSSD), both features of the time domain. Similarly, frequency domain parameters including high frequency (HF, 0.15–0.40 Hz) and low frequency (LF, 0.04–0.15 Hz) were extracted using the power spectral density of the RRI time series. The density is computed by using a Welch estimator with a Hanning window, and spectrum components are averaged by an area-under-the-curve approach.

##### 3.3.1.2 Electrodermal activity (EDA)

The wristband sensor Emotibit^2^ was used to record the activity of the sweat glands. The sensor uses wireless communication (WiFi) to stream raw EDA data at 15 Hz to a local computer connected to the same network. The sensor was placed on the non-dominant wrist of officers to avoid discomfort and facilitate data collection. The validity of EDA-based measures of the sympathetic nervous system from wrist-worn devices has been demonstrated in previous studies, showing comparable results to measured responses in the palm (Van Der Mee et al., 2021). The skin conductance level (SCL) was computed using the Neurokit2 Python libraries (Makowski et al., 2021), which reflects the overall arousal level during the session (also called the tonic phase). EDA data processing included normalization of the data and noise reduction using a high-pass filter with a 3 Hz cutoff frequency. The Emotibit sensor has been previously compared against research-grade sensors showing good levels of accuracy (Langevin et al., 2021).

#### 3.3.2 User experience and perceived usefulness of VR and physiological monitoring

##### 3.3.2.1 Virtual reality neuroscience questionnaire (VRNQ)

This questionnaire was used to measure the perceived officer’s user experience, it comprises 20 questions with a 1 to 7 Likert scale (extremely low, extremely high) covering four categories called user experience, game mechanics, in-game assistance and VR induced symptoms and effects (Kourtesis et al., 2019).

##### 3.3.2.2 Perceived usefulness

A custom-built questionnaire consisting of 6 questions related to the perception of physiological monitoring and VR training was used to capture the officers’ opinions regarding the combination of these technologies. The statement “From your perspective as a Law Enforcement Officer, what could be the most valuable aspects that could be trained using VR and physiological sensors” was followed by six aspects and a 7-point Likert scale (strongly disagree, strongly agree), exploring aspects such as self-regulation techniques, learning about body responses, unveiling potential physiological triggers, stress management skills, ideal body responses for conflict de-escalation. The questionnaire was provided before the experience as a way to identify the needs and perceived opinions of officers before being exposed to the technology.

##### 3.3.2.3 De-escalation Skills

The *De-escalating Persons in Crisis Competencies Tool* (DePICT™) is an assessment tool that measures a trainee’s demonstrated ability to de-escalate a crisis situation and highlights optimal skills integral to working with people in a mental health crisis (Lavoie et al., 2022). A collaborative community co-design approach involving a multi-perspective team of subject matter experts, including persons with lived experience of mental illness, cultural safety experts, clinicians, police instructors, and other stakeholders, was used to construct this competencies-based instrument. The DePICT™ is a 14-item rater-observer assessment scale designed to measure core competencies in the areas of risk assessment and safety; self-awareness, regulation, and person-centered approaches; communication; identifying signs and symptoms of mental illness and collaborative problem-solving. The tool provides a standardized and intuitive method of evaluating the presence of target competencies during a 7–10-min evaluation scenario that takes place in VR. The presence of each competency is rated on a 4-point scale (0 = Absent, 3 = Proficient). DePICT™ scores range from 0 to 42, where higher scores indicate higher levels of de-escalation, communication, and mental health crisis response competency acquisition. The reliability of the scale is high (Cronbach α = 0.89, *N* = 63, 14 items). A research scientist who developed the DePICT™ tool used the after-action review videos recorded in both the virtual and live environment to replay each officer’s performance during the scenario and score them. The 14 DePICT™ items comprise:

1. Risk management: Contains, manages and controls risks.
2. Time and distance: Slows situation down and gives adequate space.
3. Expresses concern for welfare: Expresses care and concern and a willingness to help.
4. Human connection: Builds rapport and personal connection.
5. Paralanguage: Conveys calming voice, tone and pitch.
6. Respectful language: Uses terms that are non-stigmatizing and respectful of the person.
7. Body language: Physically models calm behavior.
8. Self-awareness and regulation: Manages impact of own presence and regulates self-arousal.
9. Active listening: Pays attention and demonstrates listening.
10. Mental health awareness: Identifies cues of mental illness or crisis.
11. Validation: Acknowledges person’s emotions and experience.
12. External Resources: Seeks external information and resources.
13. Collaboration: Works together toward resolution.
14. Transparency: Clearly explains decisions and answers questions.

### 3.4 Procedure and data analysis

After signing the informed consent, individual officers were invited to be part of the study by completing a demographic information form. Team commanders were invited to stay while providing some context of the situation, following instructions from the researcher. Officers were instructed to respond to a dispatcher call following this script: *“Dispatch to Unit XX, we have just received a direct call from an individual at apartment 201. The caller sounded frantic, stating ‘they are trying to get in’ before disconnecting. Please proceed with caution, assess the situation, and be prepared for possible de-escalation*.” The VR head-mounted display, audio headset, and motion capture hardware were connected to the officers. The Polar chest strap sensor was connected to the officer’s chest. The Emotibit was connected to the non-dominant wrist of the officers. After connections, the Refense VR system was introduced to the officers, allowing them to go through the calibration stage and get familiar with the navigation, movement, interaction with the weapon, and communication with the dispatcher. Equipment setup, calibration, and familiarization with the Refense system lasted approximately 15 min per officer. After the calibration, officers were instructed to remain calm and steady for 3 min of standing with all the equipment to establish a physiological baseline recording. After the calibration stage, the dispatcher sent the officers a message via simulated radio indicating the start of the training. The live actor was prepared using the VR equipment to embody the character in crisis in VR. Officers completed the scenario between 7 and 12 min, after which observing team commanders were instructed to complete the DePICT™ questionnaire to evaluate the de-escalation skills of each officer. The session was stopped either when officers were able to de-escalate the situation successfully or once 12 min were completed. The session was stopped by the dispatcher and officers were asked to complete the VRNQ form. A short debrief was conducted by the team commander after completing the session, mostly focusing on the after action review recorded in the Refense system and highlighting key moments and important feedback aspects for each officer. However, no analysis was conducted of the debrief sessions.

In terms of data analysis, the physiological data processing was conducted using Python with multiple open-source libraries for the processing of physiological signals as previously described. For the statistical analysis, non-parametric tests were used for analyzing the HR and HRV parameters considering the non-normal distribution of the data. Moreover, the median values are used to describe the user experience metrics computed using the VRNQ tool. The mean values of the DePICT™ scale were used for the comparative analysis of both trainer and researcher scores.

## 4 Results

### 4.1 Physiological responses

#### 4.1.1 Cardiovascular responses: Stress regulation

**Figure 3:**
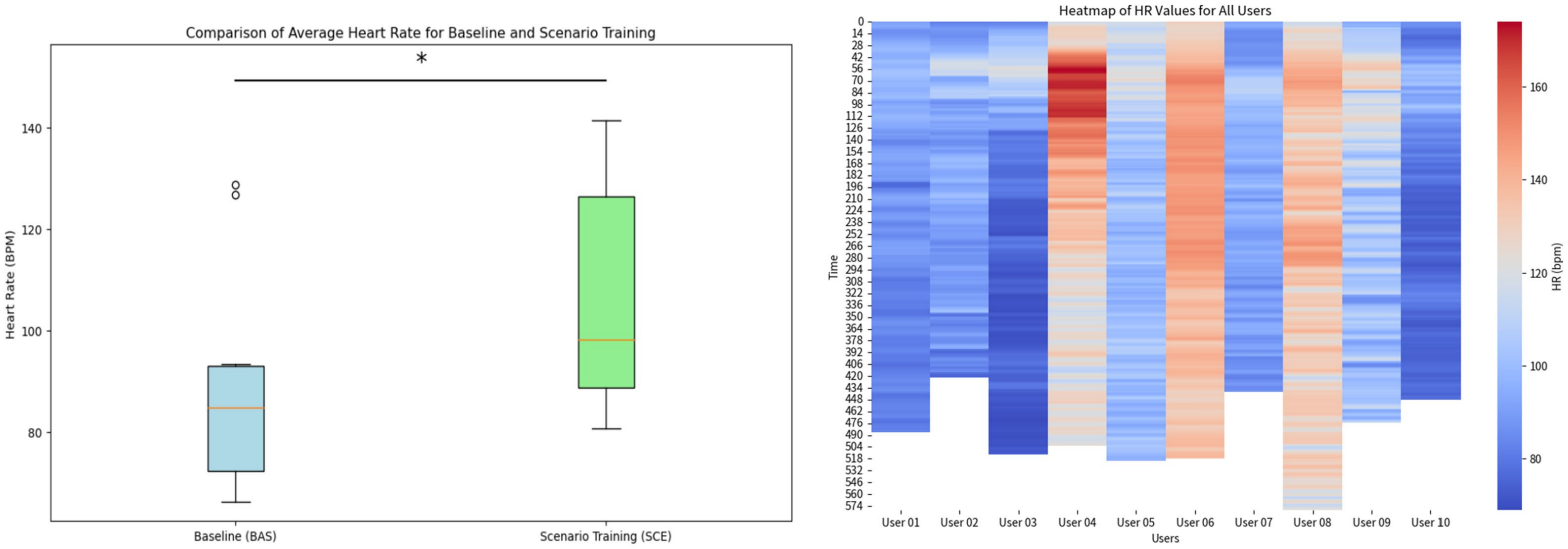
**(Left)** Boxplots representing the difference between HR level during Baseline and scenario training in VR. **(Right)** Heat maps of HR levels displaying the individual differences of the officers during the scenario training. The asterisk denotes a statistical significance of *p* < 0.05 of the difference in group average heart rate between baseline and scenario training.

Cardiovascular responses such as HR and HRV metrics associated with stress were analyzed (Kim et al., 2018). First, HR levels were compared among officers during the baseline and scenario training. Statistical analysis revealed that there was a significant difference (*p* < 0.05) in HR responses during baseline (*M* = 89 bpm, SD = 21 bpm) compared with the VR scenario training (*M* = 105 bpm, SD = 22 bpm). The boxplots visually represent the distribution of these values for each condition (see Figure 3, left). Moreover, a visualization using heat maps was created to compare the officers’ HR responses during the VR training scenario. Officers 4, 6, and 8 exhibited higher HR values compared to the rest of the officers (see Figure 3, right).

**Figure 4:**
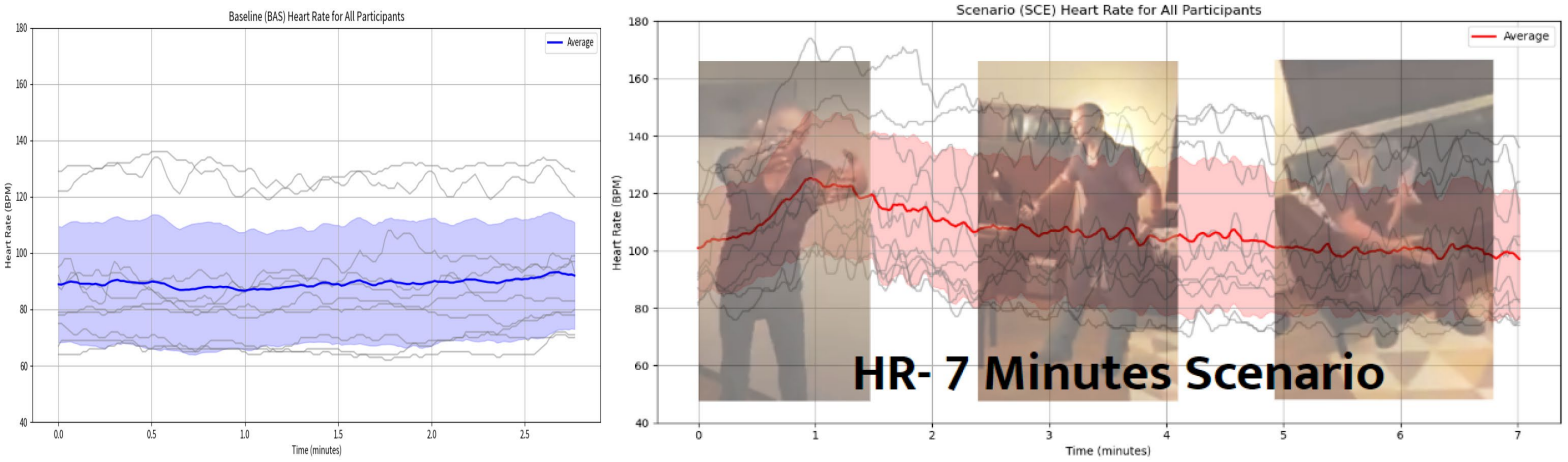
**(Left)** Average line plot of HR during baseline; gray lines represent each officer’s HR levels, the bold blue line shows the average HR and the blue shadow is the standard deviation. **(Right)** Average line plot of HR during scenario training in VR and the superimposed images show the three important moments of the scenario (arrival, escalation and de-escalation). Adapted with permission from Refense.

**Figure 5:**
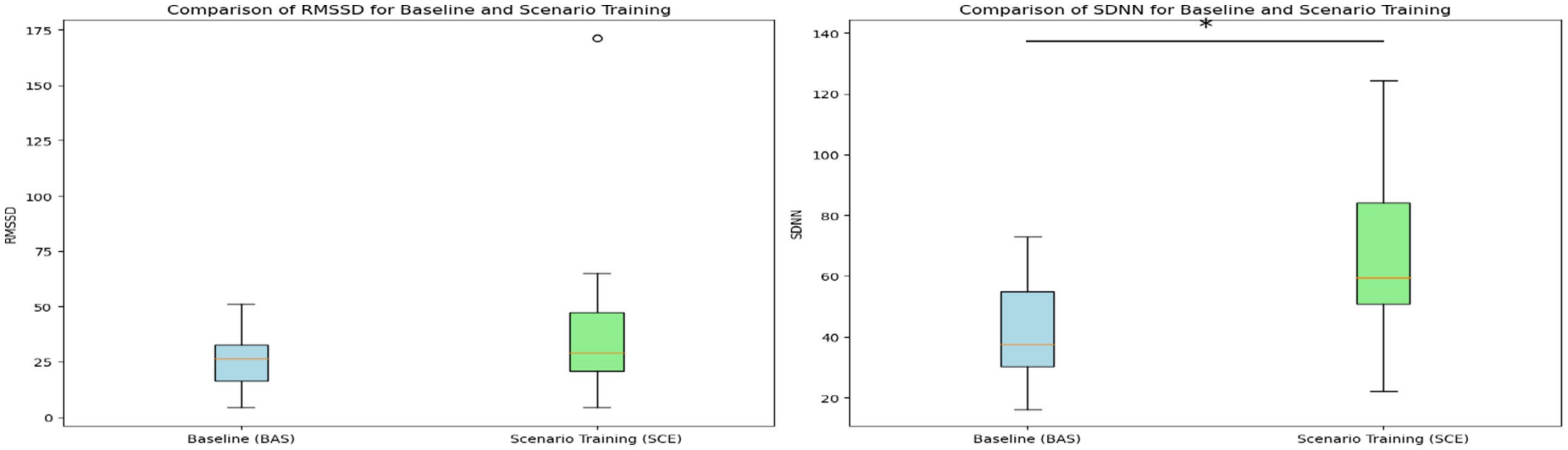
HRV analysis conducted using time domain variables. **(Left)** RMSSD values comparing baseline vs. scenario training in VR. **(Right)** SDNN values comparing baseline vs. scenario training in VR. The asterisk denotes a statistical significance of *p* < 0.05.

A visualization comparing the baseline and VR scenario training was created using aggregated data of the HR responses from the 10 officers who participated in our experiment. Figure 4 shows the HR during baseline (left) and scenario training with VR (right) for the 10 officers (gray lines), with the average line of the HR response (bold line) and the standard deviation (shadows). For the scenario training in VR, we have superimposed three images describing the three stages of the scenario and the live actor’s corresponding scripted responses. The visualization facilitates the comparison of HR responses during the baseline and the VR training (data was trimmed to the length of the shortest session to create the visualization). Additionally, HRV analysis was conducted using three variables: SDNN, RMSSD, and the ratio of LF to HF (LF/HF ratio) were computed to investigate contributions of both the sympathetic and parasympathetic nervous system during baseline and scenario training in VR. Whereas the RMSSD and the LF/HF ratio did not reveal any significant differences, the SDNN values were significantly higher (*p* < 0.05) during the scenario training in VR (*M* = 69 ms, SD = 30 ms) compared to the baseline recordings (*M* = 41 ms, SD = 17 ms) as can be seen in Figure 5. The increased variability suggests that the officers experienced dynamic shifts in their fight-and-flight (sympathetic) and their rest-and-digest (parasympathetic) activity from the baseline to the scenario training in VR.

**Figure 6:**
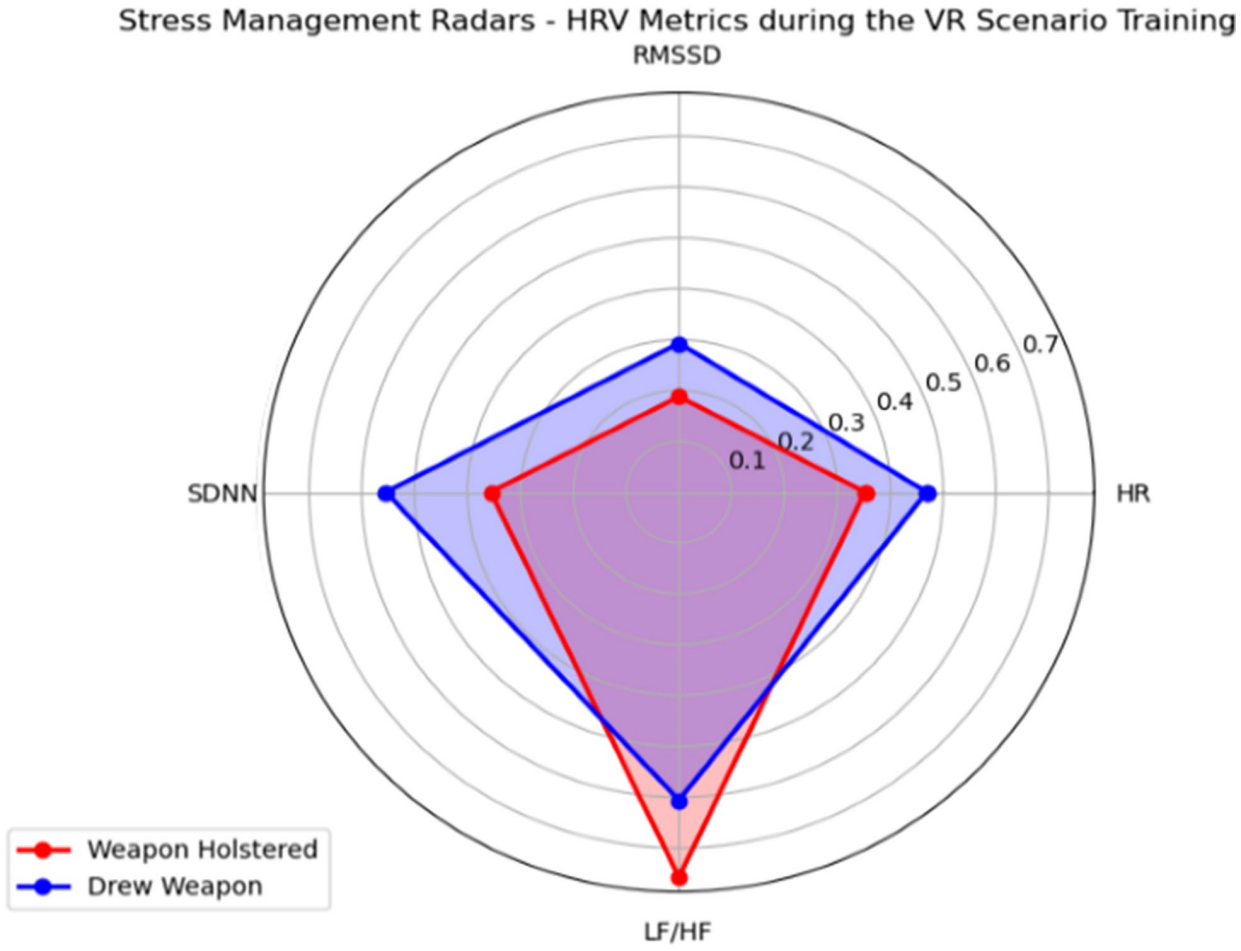
A stress management radar plot created to compare the cardiovascular responses of officers who drew the weapon with the ones who did not.

By using HRV metrics that reflect stress management skills (e.g., SDNN, RMSSD, and LF/HF ratio), we created a radar plot to compare officers who had drawn their guns and those who did not. This handling of the gun is an important behavior that both reflected and influenced the officer’s responses and the unfolding of the de-escalation scenario. A total of five users (IDs: 1, 3, 4, 8, 9) drew their guns at some point during the scenario. We clustered these two groups, normalized the values, and arranged each axis to visualize better stress management skills (e.g., the higher the axis’ value the better stress management skills). Specifically, we inverted the LF/HF ratio since typically, a higher LF/HF ratio indicates greater sympathetic activity and therefore higher stress. HRV metrics were averaged per group and the resulting polygons were overlapped in the same radar plot to facilitate the comparison. Figure 6 shows the resultant radar plot.

The radar plot revealed a “Defensive Dynamics Dichotomy” emphasizing stronger sympathetic and parasympathetic nervous system activations among officers who engaged in defensive tactics (e.g., drew a weapon) during the mental health crisis scenario. Alternatively, officers who kept their weapons holstered during the same simulation showed a more balanced response between the sympathetic and parasympathetic nervous system (e.g., greater values in LF/HF ratio), therefore suggesting comparably superior stress regulation and cognitive function.

#### 4.1.2 Electrodermal activity: physiological arousal

**Figure 7:**
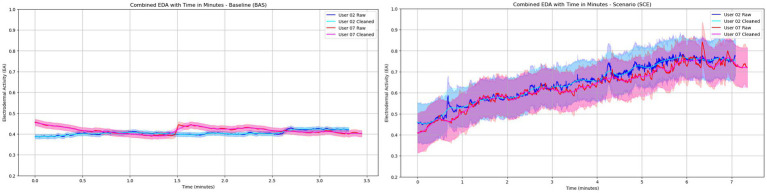
EDA patterns observed from two officers (02 and 07) depicting the baseline behavior **(left)** and the arousal responses to the VR scenario training **(right)**.

EDA data captured from the officer’s wrists was analyzed to infer physiological arousal levels. Due to multiple technical issues involving the sensor location, communication protocol (e.g., WiFi), and complexity of the setup, data from only 2 officers was used for the qualitative analysis. Baseline recordings (Figure 7, left) show EDA patterns of relaxation with a tenant slow decrease of the SCL during the 3 min of recording.

Moreover, scenario training using VR (Figure 7, right) shows a constant increase in the SCL until the end of the session, displaying a purely sympathetic activation throughout the de-escalation process. Figure 7 shows both the baseline and scenario training normalized line plots.

#### 4.1.3 User experience and de-escalation skills

##### 4.1.3.1 Virtual reality neuroscience questionnaire

**Figure 8:**
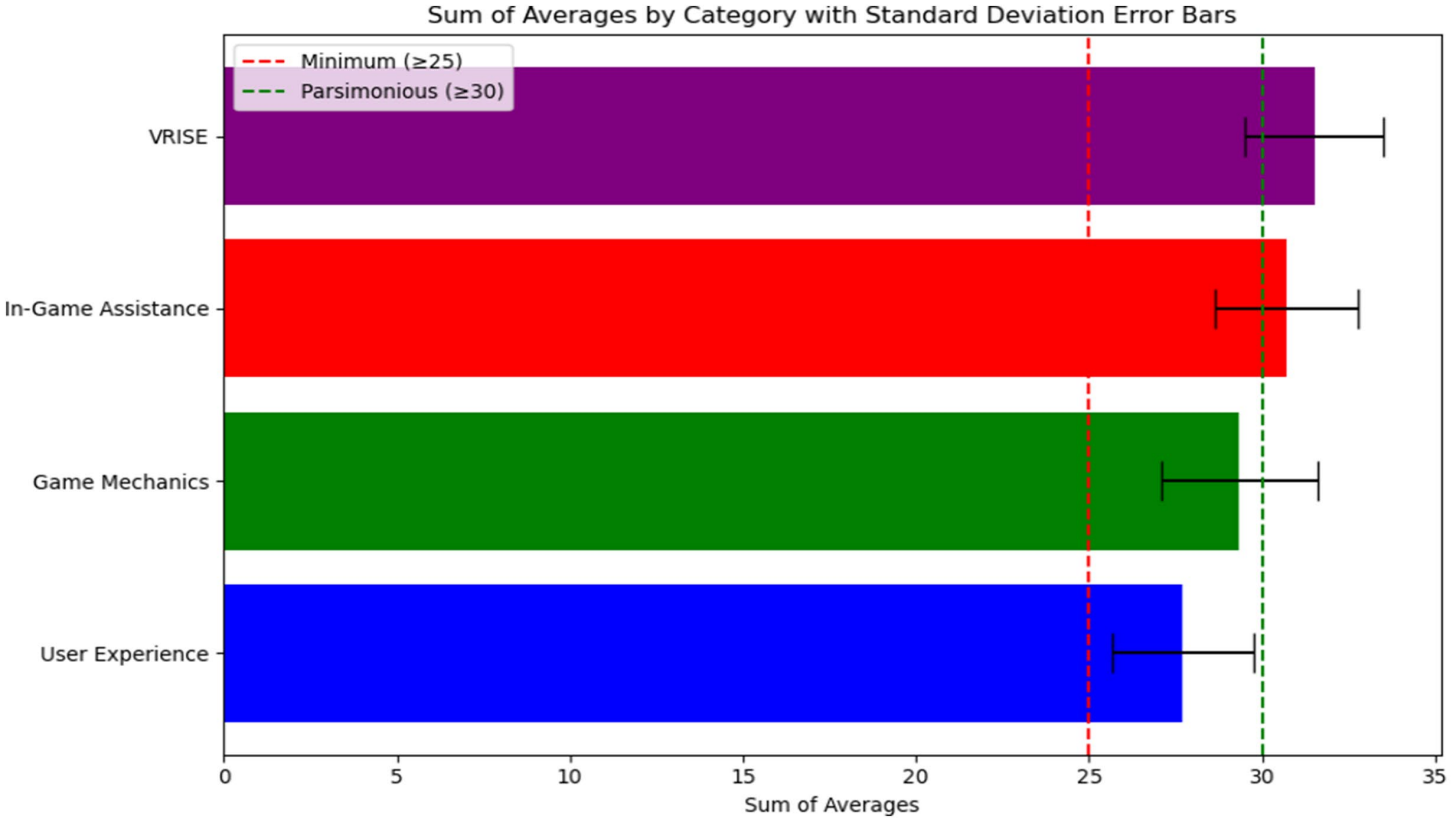
Bar plots resulting from the analysis of the VRNQ tool.

The VRNQ data was tabulated and analyzed following the four established categories: user experience, game mechanics, in-game assistance, and VRISE (VR-induced symptoms and effects). Results showed that the total of each sub-score met the minimum suggested cut-off value of 25 points (see Figure 8). This result suggests that the VR software (Refense) has adequate quality without any significant VRISE impacting the training experience. Moreover, two categories, the in-game assistance and the VRISE were found with cut-off values greater than 30 points, therefore overpassing the parsimonious cut-offs and suggesting more robustly the suitability of the VR system to create a positive experience (e.g., very mild levels of induced symptoms and tutorials and instructions perceived as highly helpful).

##### 4.1.3.2 Perceived usefulness of VR and physiological monitoring

**Figure 9:**
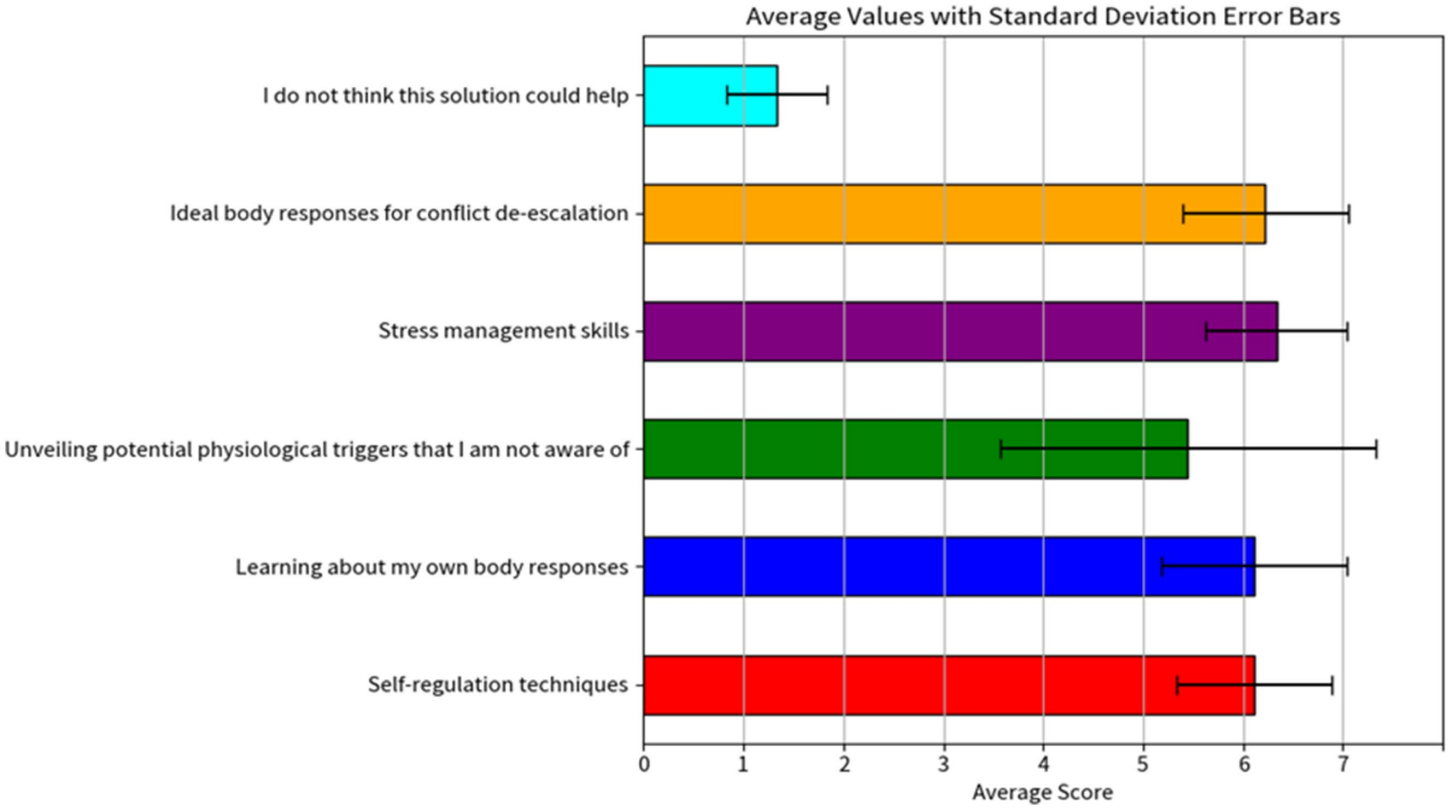
Bar plots resulting from the analysis of the perceived usefulness of VR and physiological monitoring technologies tool.

This custom-built questionnaire showed that among the 10 police officers, there was an overall agreement that physiological monitoring technologies (e.g., chest strap, wristband) were useful as a tool to improve their training skills (see Figure 9). Police officers perceived physiological monitoring technologies as particularly valuable as a tool for stress management and identification of body responses associated with effective conflict de-escalation.

##### 4.1.3.3 De-escalation skills using DePICT™

**Figure 10:**
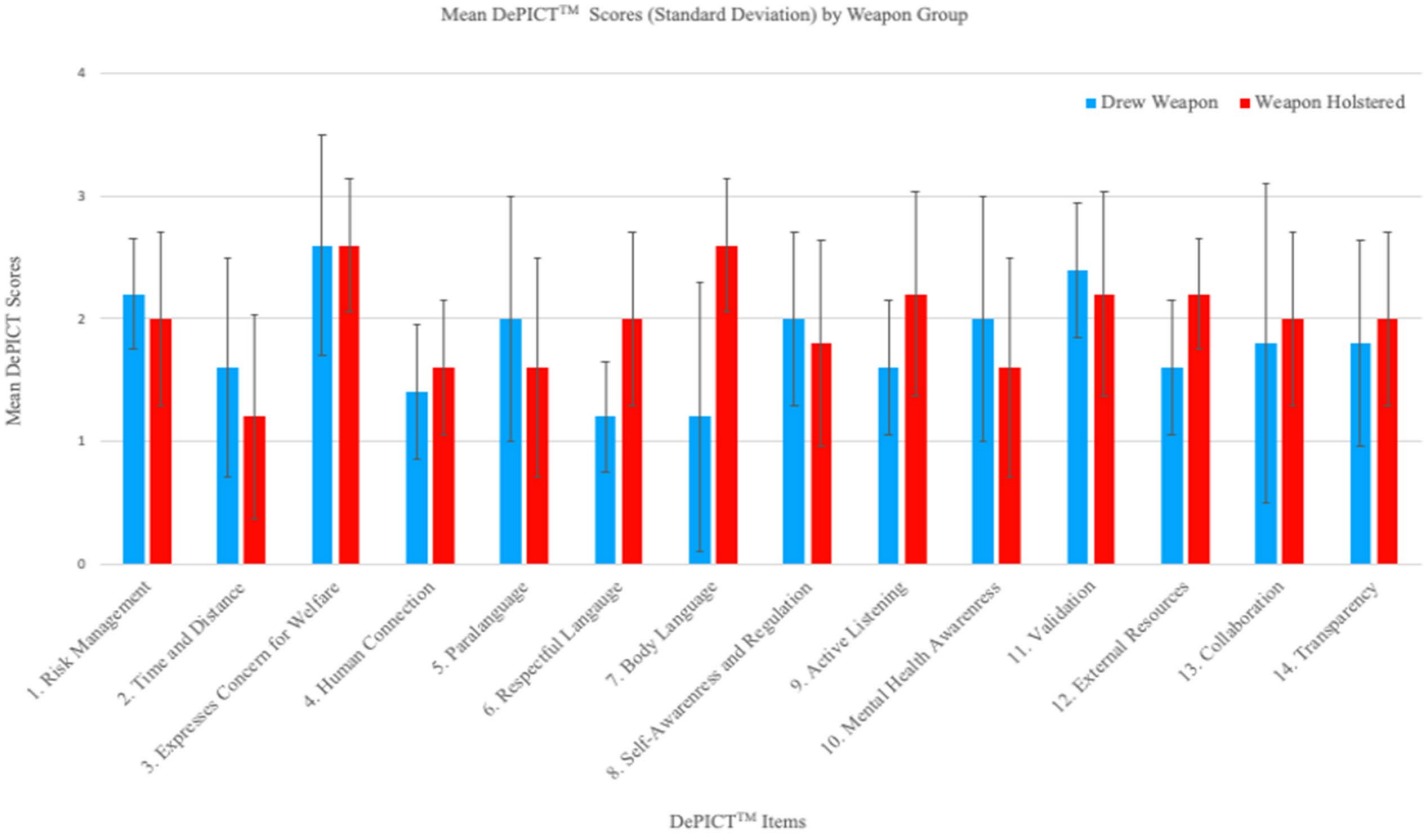
DePICT™ scale bar graph showing the differences between the scores of officers who drew the weapon and those who did not.

In the analysis of tactical communication and de-escalation techniques, the bar plot of the DePICT™ scale (Figure 10) reveals overall high scores in expressing concern for welfare and a willingness to help, validating the emotions of the person in crisis, and approaching, containing and controlling the scene for effective risk management. The average DePICT™ total score for the sample (*n* = 10) was *M* = 26.5, SD = 5.73 out of a total score of 42. Officers who did not draw a weapon had a slightly higher mean DePICT™ total score (*M* = 27.6, SD = 6.50) relative to officers who drew a weapon during the simulation (*M* = 25.4, SD = 5.73). Contrarily, management of time and distance, promoting a human connection, and using respectful and non-stigmatizing language showed the lowest average scores among the officers. A unique pattern of de-escalation competencies was revealed among officers who relied on weapons resolving the scenario relative to those who did not. Officers who kept their weapons holstered showed marked elevations in both verbal and non-verbal de-escalation techniques such employing calming body language, such as using an open stance (item 7), using non-stigmatizing and respectful language (item 6), using active listening techniques, such as paraphrasing (item 9), and resourcefulness in seeking additional information from other sources and leveraging external units.

Alternatively, officers who drew a weapon were marginally more likely to focus on officer safety tactics, such as risk management (item 1) and increasing time and distance from the person in crisis (item 2). They were also slightly more likely to recognize and acknowledge the presence of mental health issues (item 10) and use calming paralanguage to validate the distressed emotions of the person in crisis.

## 5 Discussion

Evidence suggests that police officers receive insufficient training in responding to mental health calls for service and have training gaps in fundamental de-escalation skills (Lorey and Fegert, 2021). Adequately designed training is necessary for developing the best possible practices in policing and conflict de-escalation when addressing individuals with mental health disorders. The findings of this research provide valuable insights into the effectiveness of VR training for MHCR in active police officers and contribute to the development of evidence-based training programs supported with advanced VR technology (Starcke and Brand, 2012). We have investigated the physiological sympathetic activation of a group of officers who experienced a stress-inducing scenario in VR involving a live actor and multiple wearable technologies to create a highly realistic scenario of an MHCR call.

### 5.1 Physiological interpretation and sympathetic activation

The statistical analysis revealed that there was a significant difference in HR responses during baseline compared with the VR scenario training. The pictures overlapping the HR responses are designed to facilitate the interpretation of key moments during the training by indicating the cardiovascular changes of the police officers during the different stages of de-escalation as revealed during the VR scenario. Higher levels of baseline or resting HR during engagements in violent encounters of active law enforcement officers in the USA were associated with an increased risk of officers engaging in a violent altercation (Fox et al., 2018). Lower HR levels during stressful moments are normally associated with better decision-making skills among police officers. Studies have shown that physiological arousal, as measured by heart rate, increases during police encounters, particularly during high-priority calls (Baldwin et al., 2019; Andersen et al., 2020). However, HR alone is not a reliable predictor of officer performance, during sympathetic activation, HR and respiration increase, and police officers are more likely to hold their breath, limiting the amount of oxygenated blood coming to the brain (Andersen and Gustafsberg, 2016). Similar studies have highlighted the significant impact of stress on officer performance in critical situations and the need for effective stress management training, even for experienced officers, suggesting that more nuanced measures of stress reactivity beyond just HR may be important for assessing readiness and performance potential (Baldwin et al., 2022). Repeated exposure to stressful situations can also increase HR, highlighting the importance of allowing de-escalating rests between performing tasks. Therefore, while elevated HR may not necessarily predict performance in police decision-making, it is likely to be helpful for officers to be aware of their physiological arousal and use that information to help control their emotions during high-stress situations. VR scenario-based training can be a catalyst for this awareness and control by allowing police to be exposed to multiple stressful scenarios repeatedly. Moreover, de-escalation training programs could leverage VR technologies coupled with physiological monitoring devices to guide officers in defusing critical incidents and help improve their ability to de-escalate by self-modulating their physiological responses (Zahabi and Abdul Razak, 2020). The results are consistent with the literature, showing the physiological signatures of stress and its impact on police officers’ duties, especially connecting how elevated HR from critical moments could remain throughout for minutes and even hours after the occurrence (Baldwin et al., 2019). The literature on police performance seen through the lenses of cardiorespiratory responses, shows that, in individuals who experience threat-like situations, the blood circulation is restricted in the periphery due to task demands exceeding their coping resources (Kelley et al., 2019).

The visualization comparing the baseline and VR scenario training showed that the HRV (SDNN) values were significantly higher during the scenario training in VR compared to the baseline recordings. SDNN is normally associated with the modulation of both the sympathetic and parasympathetic nervous systems, which has been found crucial for important decision-making skills for police officers training (Di Nota et al., 2021). This finding is consistent with the fear or sympathetic activation theories in psychophysiology and neuroscience, which suggest that the sympathetic nervous system is activated during stressful situations, leading to variations of HRV metrics and other physiological responses (Brisinda et al., 2015). Moreover, HRV biofeedback training is a powerful self-regulation technique that could be integrated into modern VR simulations for police training, creating opportunities for more physiologically aware training and potentially enhancing decision-making and situational awareness (Brammer et al., 2021).

Moreover, radar plots were used to explore HRV and stress management differences among officers who drew a gun during the simulation and those who always kept the gun holstered. The LF component of the LF/HF ratio is often linked to both sympathetic and parasympathetic nervous system activity, while the HF component is predominantly influenced by sympathetic activity (Kim et al., 2018). The LF/HF ratio is thus considered a marker of the balance between sympathetic and parasympathetic influences (also called autonomic regulation), with a higher ratio indicating a shift toward sympathetic dominance and a lower ratio indicating a shift towards parasympathetic dominance. Since we inverted the LF/HF axis to reflect better stress management skills in the radar plot, we interpret that when an officer draws a gun, it generally indicates a heightened state of alertness or preparedness for a potential threat. This action leads to increased sympathetic nervous system activation, reflected in a higher LF/HF ratio, suggesting higher sustained stress levels. On the other hand, not drawing a gun might indicate a lower perceived threat level, leading to lower stress levels and a more balanced or parasympathetic-dominant state, reflected in a lower LF/HF ratio. In the context of stress management, officers who did not draw their guns might have better stress management skills to face the stressors in the training scenario. The possible better skills are indicated by their lower LF/HF ratios (reflected as a higher value in the radar plot), suggesting they were able to maintain a calmer and more collected physiological state. Better stress management skills could be beneficial for decision-making and cognitive function, as excessive stress can significantly impact these abilities in real-life scenarios. Similar results were found in a recent review of stress monitoring in first responders and tactical operators using HRV, where the LF/HF ratios were found to change during stressful moments with reported suppressions of LF and HF components due to sympathetic and parasympathetic shifts (Corrigan et al., 2021). Nevertheless, it is worth noticing that other phenomenological aspects of stress management could be used to explain changes in the LF/HF ratio. For instance, officers who drew their weapons likely experienced higher anticipatory stress, anticipating potential confrontation. This heightened state of alertness would result in increased sympathetic activation and a lower LF/HF ratio. On the other hand, officers who maintained a holstered weapon may have encountered fewer immediate stressors, leading to a more balanced autonomic response (Tornero-Aguilera et al., 2018).Additionally, the results from the EDA analysis showed a sustained increase, with the conductance level exhibiting an upward pattern, illustrating that the stressor does not wear off throughout the simulated scenario (Boucsein, 2012). This pattern has been associated with situations where stressors are intense and users are constantly in a permanent state of alertness and physiologically aroused (Kelley et al., 2019), therefore clearly displaying a strong activation of the sympathetic nervous system. Moreover, the simulation was effective in keeping officers attentive and aroused during the entire scenario since, contrary to conventional wisdom, EDA tends to decrease with very stressful or frustrating situations and this has been proven already in VR environments (van Dammen et al., 2022).

In summary, both cardiovascular and electrodermal responses recorded showed unique traces of both sympathetic and parasympathetic nervous system activity during the MHCR training scenario, demonstrating the capabilities of immersive VR to evoke measurable physiological responses in active police officers.

### 5.2 User experience and de-escalation skills

Overall police officers found the VR experience to be enjoyable and elements such as in-game assistance and VR-induced symptoms and effects were perceived positively after the VR training. Aspects related to full-body immersion, the use of weapon replicas, and the realism of the scenario involving the actor, created a very compelling simulation for MHCR training. A comparison across multiple commercially available VR systems in Europe showed that systems like the one used for this experiment (e.g., Refense), offer a high level of immersion and acceptance across police departments due to the advanced technical features such as integration with motion capture technologies and the after-action-review.^3^ Although little evidence exists around the overall acceptance of police officers and trainers over the use of VR, especially for MHCR training, it is evident that interactive and immersive technologies such as VR will transform the conventional training methodologies, creating new tools for training important de-escalation skills such as empathy and interpersonal skills (Xie et al., 2021; Lavoie et al., 2023). Jenkins and colleagues recommend that researchers move beyond shoot/no shoot scenarios where the correct decision is apparent towards more ambiguous and complex situations, such as the scenario used in the current study featuring a person in crisis armed with a weapon of opportunity. Progressing to the use of “gray” scenarios is valuable not only for training officers to better de-escalate interactions with the public, but also for disentangling the unique effects of an officer’s training and/or personality on performance. For instance, “stress-based training,” which focuses on exposing officers to physically demanding, high-stress scenarios and training them to respond with vigilance, defensive tactics, force and weaponry may nurture a “warrior” orientation (McLean et al., 2020; Li et al., 2021). It has been argued that such a mindset may prime officers to perceive non-compliant or disordered behaviors as more dangerous than they are, leading to the use of unnecessary force (Li et al., 2021). Insights using complex crisis scenarios could lead to developing safeguards against the use of excessive force or inappropriate deadly force in mental health crisis situations. Moreover, we also evaluated the officer’s perception of physiological monitoring technologies and discovered an overall high usefulness perception in areas such as stress management and self-regulation techniques. These results are similar to the ones found in a recent study evaluating active police officers’ perception of wearable technology to improve their awareness and understanding of sleep, well-being, and quality of life (Cox et al., 2024). Particularly, metrics of HRV and sleep quality and recovery were perceived as a very valuable (Cox et al., 2024).

On the other hand, officers 5 and 6 showed the highest DePICT™ total scores (both scoring 34 respectively), suggesting they demonstrated strong de-escalation skills. They also have relatively high HRV values, indicating a well-managed stress response during the VR scenario. This combination of high DePICT™ scores and high HRV suggests they performed best in terms of both de-escalation competency and physiological stress regulation.

In considering the patterns revealed in Figure 6, it is reasonable to conclude that officers who elect to draw weapons (e.g., an escalating action) might engage in additional necessary de-escalation strategies compared to officers whose weapons remained holstered. Interestingly, these results suggest that the display of force (in this case, drawing or pointing a service pistol) is not wholly inconsistent with de-escalation efforts. Consistent with the radar plot showing that officers who relied on a weapon experienced stronger sympathetic and parasympathetic nervous system responses and lowered balanced stress management and cognitive function, these same officers demonstrated commendable DePICT™ skills. First, officers who relied on defensive tactics to resolve the scenario were more likely to focus on officer safety techniques, including containing and controlling risks, slowing down the encounter, and increasing distance from the person in crisis. These actions signal that the officer may have perceived the person in crisis as threatening, explaining sympathetic system activation. Given that these same officers were also more likely to recognize the presence of mental health issues, a threatening perception may have been bolstered through the potential activation of mental illness-related stereotypes linked to dangerousness and unpredictability (Corrigan and Watson, 2005). The marginally higher DePICT™ scores on self-regulation efforts and self-awareness of their authority are also consistent with the radar plot showing that officers who drew weapons oscillated to stronger parasympathetic nervous system responses (as seen by higher values of RMSSD) following strong fight/flight activation. It is possible that lower cognitive functioning demonstrated among officers who drew a pistol may be explained by either (i) experience of fear or (ii) the requirement to continuously evaluate legal/policy adherence and liability implications connected to displaying a firearm. We found that officers who demonstrated better stress management as evidenced on the radar plot, did not use as diverse de-escalation strategies. Instead, they differentially relied on communication techniques such as actively listening, using respectful language, and calming body language to bring the situation to a resolution without the use of force. These disparities suggest differing perceptions or emphases on certain techniques, highlighting areas where training and operational protocols may need alignment or further development. The directionality of these effects remains to be tested, specifically, these patterns must be deconstructed to understand the sequencing of effects among officers who engage in defense tactics. Overall, the analysis underscores the necessity of consistent training and evaluation methods in de-escalation and communication for crisis management, ensuring a unified and effective approach.

The observed shifting between the sympathetic and parasympathetic activation during the VR simulation training can be explain with what we call a “Defensive Dynamics Dichotomy,” which highlight significant differences in autonomic responses between SWAT officers who engage in defensive actions, such as drawing a weapon, and those who do not. This dichotomy underscores the critical importance of stress management in tactical performance. Officers engaging in defensive actions exhibited heightened sympathetic nervous system activity, reflected in increased HRV and EDA, indicating elevated stress levels. In contrast, those who did not engage in such actions maintained more stable autonomic regulation, demonstrating better stress management and decision-making capabilities under pressure. These findings suggest that integrating stress management techniques and real-time physiological monitoring into training protocols can enhance officers’ ability to regulate their autonomic responses, thereby improving their performance and decision-making in high-stress situations (Andersen et al., 2018).

Future research should focus on several key areas to enhance our understanding of physiological and behavioral responses in high-stress law enforcement scenarios in VR. First, it is essential to confirm distinctive physiological signatures between officers who kept their weapon holstered versus those who drew it, providing clearer insights into stress regulation and decision-making processes. Additionally, comparing patrol officers with SWAT officers could reveal significant differences in stress responses and tactical approaches, given their distinct training and operational environments (Jenkins et al., 2024). Incorporating additional physiological measurements, such as electroencephalography (EEG) (Johnson et al., 2014) and affective measurements (e.g., facial electromyography – fEMG), could further explore aspects of empathy and emotional regulation. Finally, gathering more qualitative data, including detailed debriefing sessions with both officers and instructors, will offer valuable perspectives on the subjective experiences and practical implications of different stress management strategies. This comprehensive approach will contribute to developing more effective training programs and improving law enforcement practices. Some of the important limitations identified for this study can be summarized as follows:

- Small sample size: ten active police officers with SWAT training can be considered as a small cohort for these studies. This may not adequately represent the broader population of law enforcement personnel, particularly those without specialized SWAT training or those from diverse geographical and departmental backgrounds. The limited sample size restricts the statistical power of the findings, making it challenging to generalize the results to the wider law enforcement community. Nevertheless, it is important to consider that recruiting active police officers for de-escalation training studies is a common challenging task considering the shortage of personnel and lack of availability of police officers in many departments and the novelty of the VR training.
- Uniform and scenario mismatch: the use of SWAT uniforms in a scenario designed for patrol services may not accurately represent the typical attire and equipment patrol officers would use in a mental health crisis response. This discrepancy could influence the physiological responses and decision-making processes of participants, potentially limiting the applicability of findings to patrol officers in real-world scenarios. Future studies should aim also to include patrol officers to accurately represent the population the scenario was built for.
- Ethnicity representation in VR: the ethnicity of the live actor (male African American) differed from the avatar’s ethnicity (white male) presented in the VR scenario. This inconsistency may affect the officers’ perception and interaction within the simulation, introducing a variable that could skew the interpretation of physiological responses and de-escalation strategies employed by participants.
- EDA data analysis: due to some technical difficulties, the EDA sensor corrupted many of the participant’s data, impeding a more comprehensive analysis of this important physiological marker. This situation certainly reveals one of the most common issues in physiological monitoring: the challenge of connecting multiple wearable sensors, transmitting using different wireless protocols (e.g., Wifi vs. Bluetooth) in already complex VR simulation scenarios. We believe the WiFi of the Motion Capture cameras interfered the streaming of the Emotibit data, then corrupting the signals.

These limitations highlight the need for careful consideration of scenario design, participant equipment, and representation within VR simulations to ensure they accurately reflect the intended training contexts. Also, the importance of carefully exploring data communication protocols and sensors during police training to partially prevent issues with data collection. Addressing these limitations in future studies could enhance the fidelity of VR training environments and provide more generalizable insights into the psychophysiological effects of VR training on law enforcement officers.

## 6 Conclusion

This study investigates the effects of VR training for police officers, particularly those with SWAT training, underscoring the significant psychophysiological impacts of immersive VR scenarios on stress regulation, arousal levels, and decision-making processes. By HR and HRV, this research highlighted the enhanced stress response and cognitive function under a very stressful simulated de-escalation scenario, offering insights into the physiological dynamics of law enforcement personnel during mental health crisis interventions. A key finding, the “Defensive Dynamics Dichotomy,” indicates differing autonomic responses between officers who engaged in defensive actions, such as drawing a weapon, and those who did not, pointing to the critical role of stress management in effective tactical performance. This study also revealed positive feedback from active police officers regarding the VR training experience, suggesting minimal adverse effects and a general acceptance of VR as a valuable training tool. This acceptance is critical for the adoption of VR in enhancing police training outcomes, particularly in developing crucial de-escalation skills in a controlled setting. Broader implications of this research suggest the potential of VR and physiological monitoring technologies in creating evidence-based training programs that can fill current gaps in police training, especially in responding to mental health crises. Such programs aim to improve law enforcement training methodologies, prepare officers more effectively for real-life encounters, and ensure safer interactions with the public. Overall, our contributions help validating the effectiveness of VR combined with physiological monitoring technologies as a potent training medium in law enforcement, capable of producing measurable physiological and psychological responses. It encourages ongoing exploration into VR’s potential in expanding police training frameworks to encompass a wider variety of scenarios and participants, ultimately enhancing law enforcement capabilities and public safety outcomes.

## Data availability statement

The dataset presented in this article is not readily available due to privacy requirements of the involved police department. Requests to access the datasets should be directed to john.munoz.hci@uwaterloo.ca.

## Ethics statement

This study involving human subjects was approved by the University of Waterloo ERB, (ORI: #44796) and the Wilfrid Laurier University ERB (ORI: #8925). The studies were conducted in accordance with the local legislation and institutional requirements. The participants provided their written informed consent to participate in this study. Written informed consent was obtained from the individual(s) for the publication of any potentially identifiable images or data included in this article.

## Author contributions

JM: Conceptualization, Data curation, Formal analysis, Investigation, Methodology, Software, Validation, Visualization, Writing – original draft, Writing – review & editing. JL: Conceptualization, Data curation, Formal analysis, Funding acquisition, Investigation, Methodology, Resources, Software, Writing – original draft, Writing – review & editing. AP: Conceptualization, Formal analysis, Investigation, Methodology, Visualization, Writing – original draft, Writing – review & editing.

## Footnotes

^1^ https://www.refense.com/

^2^ https://www.emotibit.com/

^3^ https://shotpros.eu/wp-content/uploads/2021/01/SHOTPROS_Booklet_VR-Provider_V1.pdf
